# Relationship between clinical phenotype and in vitro analysis of 13 NPT2c/SCL34A3 mutants

**DOI:** 10.1038/s41598-022-25995-5

**Published:** 2023-01-03

**Authors:** François Brazier, Marie Courbebaisse, Amandine David, David Bergerat, Christine Leroy, Marta Lindner, Gérard Maruani, Camille Saint Jacques, Emmanuel Letavernier, Marguerite Hureaux, Rosa Vargas-Poussou, Dominique Prié

**Affiliations:** 1 Université de Paris Cité, Faculté de Médecine, INSERM U1151, Paris, France; 2 Département de Physiologie, hôpital Necker Assistance Publique Hôpitaux de Paris, Paris, France; 3 Service de Physiologie, hôpital européen Georges Pompidou, Assistance Publique Hôpitaux de Paris, Paris, France; 4 Service de Génétique, hôpital européen Georges Pompidou, Assistance Publique Hôpitaux de Paris, 75015 Paris, France; 5 Service d’Explorations fonctionnelles multidisciplinaires, hôpital Tenon, Assistance Publique Hôpitaux de Paris, Paris, France; 6 Université Paris Sorbonne, Paris, France; 7 Université de Paris Cité, Faculté de médecine, INSERM U970, Paris, France

**Keywords:** Physiology, Kidney, Nephrons, Genetics, Medical genetics

## Abstract

Biallelic pathogenic variants in the *SLC34A3* gene, encoding for the NPT2c cotransporter, cause Hereditary Hypophosphatemic Rickets with Hypercalciuria (HHRH). However, the associated phenotype is highly variable. In addition, mice deleted for *Slc34a3* exhibit a different phenotype compared to humans, without urinary phosphate leakage. The mechanisms by which *SLC34A3* variants disrupt phosphate/calcium metabolism are un-completely understood. In this study we explored these mechanisms in vitro using *SLC34A3* variants identified in patients with urinary phosphate leakage. We analyzed the consequences of these variants on NPT2c function and the link with the phenotype of the patients. We studied 20 patients with recurrent nephrolithiasis and low serum phosphate concentration harboring variants in the *SLC34A3* gene. Half of the patients carried homozygous or composite heterozygous variants. Three patients had in addition variants in *SLC34A1* and *SLC9A3R1* genes. All these patients benefited from a precise analysis of their phenotype. We generated 13 of these mutants by site-directed mutagenesis. Then we carried out transient transfections of these mutants in HEK cells and measured their phosphate uptake capacity under different conditions. Among the 20 patients included, 3 had not only mutations in NPT2c but also in NPT2a or NHERF1 genes. Phosphate uptake was decreased in 8 NPT2c mutants studied and normal for 5. Four variants were initially categorized as variants of uncertain significance. Expression of the corresponding mutants showed that one did not modify phosphate transport, two reduced it moderately and one abolished it. Co-transfection of the NPT2c mutants with the wild-type plasmid of NPT2c or NPT2a did not reveal dominant negative effect of the mutants on NPT2c-mediated phosphate transport. A detailed analysis of patient phenotypes did not find a link between the severity of the disorder and the level of phosphate transport impairment. NPT2c mutations classified as ACMG3 identified in patients with renal phosphate leak should be characterized by in vitro study to check if they alter NPT2c-mediated phosphate transport since phosphate uptake capacity may not be affected. In addition, research for mutations in NHERF1 and NPT2a genes should always be associated to NPT2c sequencing.

## Introduction

In 1985, a rare syndrome associating hypophosphatemia and rickets was described in a large inbred Bedouin tribe and was called Hereditary Hypophosphatemic Rickets with Hypercalciuria (HHRH)^1^. Affected patients have rickets and/or osteomalacia but also low serum phosphate and a decrease in the ratio of maximum tubular reabsorption of phosphate to glomerular filtration rate^2^. This form of rickets is characterized by a pronounced hypercalciuria. The biological abnormalities observed in these patients are similar to those described in patients with a heterozygous mutation of *SLC34A1* and in mice with a homozygous deletion of *Slc34a1*^3,4^. In the HHRH patients, hypercalciuria is associated with high 1,25(OH)_2_D serum concentration and low FGF23 level in the plasma. Bone morphological abnormalities may be associated with bone pain but also muscle weakness. The elevation of 1,25(OH)_2_D concentrations causes a decrease in PTH which may contribute to increase urinary calcium excretion. Genetic studies on various families with HHRH identified the locus associated with the disease, and then showed that biallelic pathogenic variants in the *SLC34A3* gene, that encode NPT2c protein, were the cause of the phenotype^5^.

However, subsequent studies of patients harboring *SLC34A3* biallelic pathogenic variants showed a large diversity of the phenotype, low serum phosphate concentration and rickets being inconstant. Hypercalciuria can be the sole anomaly observed in some patients. Hypercalciuria is thought to be secondary to elevated calcitriol serum concentration and could generate urinary lithiasis^6^. Therefore, patient phenotype varies a lot, ranging from the absence of urinary phosphate loss to recurrent urolithiasis, nephrocalcinosis or hypophosphatemic rickets^4^.

Mice with targeted deletion of *Slc34a3* gene do not exhibit hypophosphatemia, high phosphate excretion in urine, renal calcification, rickets or osteomalacia^7^. Their phenotype associate hypercalcemia, hypercalciuria, a rise in 1,25(OH)_2_D and a decrease in FGF23 serum concentrations. NPT2a protein was not overexpressed in the kidney which could not explain the lack of low serum phosphate concentration^8^.

In order to better understand the responsibility of *SLC34A3* pathogenic variants in the phenotype of patients with renal phosphate leak or recurrent urolithiasis, the aim of the present study was to analyze the consequences of 13 identified *SLC34A3* variants on NPT2c phosphate transport capacity.

## Results

### Patients phenotype

Patients were included between 2003 and 2014. During this period, genetic exploration for *SLC34A1* (encoding NPT2a), *SLC34A3* (encoding NPT2c) and *SLC9A3R1* (encoding NHERF1) was performed in 245 patients with either kidney stone disease or urinary phosphate leakage. 173 patients had no genetic sequence variation in the genes studied. Among the 72 remaining, 20 had a family history of lithiasis disease, these patients were included in this study. The phenotypic and genetic characteristics of these 20 unrelated patients are presented in Tables 1 and 2. At the time of the diagnosis the patient mean age was 46.7 ± 16.4 years (min–max: 15–72 years of age). Five patients received vitamin D and one oral phosphorus supplementation. At the time of the exploration, none of the patients had calcium supplementation and their other treatments were not discontinued. Serum calcium concentration was normal in all patients. The median estimated GFR was 83 ml/min/1.73 m^2^. Apart from patient #18 whose estimated GFR was 55 ml/min/1.73 m^2^, none of our other patients had significant renal impairment (eGFR < 60 ml/min/1.73 m^2^). Fifty percent of patients had hypercalciuria defined by a calcium/creatinine ratio higher than 0.55 mmol/mmol and 60% had elevated serum calcitriol concentration (> 60 pg/ml for adults; > 120 pg/ml for adolescents^9^). All patients had low serum phosphate concentration (< 0.85 mmol/l in adults and < 1.3 mmol/l in teenagers) and low renal phosphate reabsorption (TMPO4/GFR < 0.70 mmol/l) or low TRP (< 80%). PTH concentration was below the threshold of detection in 2 patients and was low at 9 pg/ml in another patient. None had significant hyperparathyroidism. 7 patients had increased plasma concentrations of osteocalcin and/or bone alkaline phosphatase and/or collagen C telopeptide 1, indicating increased bone remodeling (Table 1). Two patients had rickets and two patients had osteoporosis. Serum full length-FGF23 concentration measured in 5 patients was within normal values (Table 1). We analyzed by infrared spectrometry the kidney stones of 7 of our patients. For 6 of the 7 patients, the major component was composed of either whewellite or weddellite. For a single patient, the major component was carbapatite. Ultrasound or CT scan revealed the presence of one or two renal cysts in 4 patients.

**Table 1:** Phenotypic characteristics of patients with SLC34A3 variants.

Various				Clinical context		Laboratory tests				
Patient Id	Age at the time of exploration (years)	Height (cm)	Gender	Mineral metabolsim related treatment	Medical background	Serum phosphate (mmol/l)	TmPO4/GFR (mmol/l GFR)	TRP (%)		
1	64	147	F	None	Recurrent nephrolithiasis; osteoporosis	0,82	0.6/0.81^a^	–		
2	63	162	F	None	Recurrent nephrolithiasis	0,69	0.54	–		
3	56	167	M	None	Recurrent nephrolithiasis	0,74	NA	62		
4	58	162	M	None	Recurrent nephrolithiasis	0,68	NA	51		
5	46	171	M	None	No specific medical history	0,56	0.37	–		
6	35	NA	F	Vitamin D	Recurrent nephrolithiasis	0,76	0.54	–		
7	16	NA	M	None	Recurrent nephrolithiasis	0,80	NA	75		
8	33	179	M	None	Mild nephrolithiasis	0,73	0.56	–		
9	50	159	F	None	Recurrent nephrolithiasis	0,74	0.47	–		
10	15	143	F	Vitamin D; phosphorus	Mild nephrolithiasis; rickets	1,27	NA	61		
11	54	159	F	Vitamin D	Recurrent nephrolithiasis	0,66	0.54	–		
12	38	157	F	None	Recurrent nephrolithiasis	0,68	0.55	–		
13	27	169	M	None	Nephrocalcinosis; rickets	0,72	0.51	–		
14	40	168	M	None	Recurrent nephrolithiasis; nephrocalcinosis	0,53	0.58	–		
15	52	184	M	None	Recurrent nephrolithiasis	0,69	0.51	–		
16	68	166	M	Vitamin D	Recurrent nephrolithiasis; osteosclerosis	0,63	NA	70		
17	36	177	M	Vitamin D	Recurrent nephrolithiasis	0,79	0.65	–		
18	62	180	M	None	Recurrent nephrolithiasis	0,69	0.68	–		
19	72	171	M	None	Mild nephrolithiasis; osteoporosis	0,80	NA	81		
20	49	175	M	None	Recurrent nephrolithiasis	0,71	0.57	–		

Various	Laboratory tests									
Patient Id	Serum calcium (mmol/l)	Fasting Uca/Ucr (mmol/mmol)	25OHD (ng/ml)	1,25(OH)2D (pg/ml)	PTH (pg/ml)	MDRD eGFR (ml/min/1.73 m^2^)	OCN (ng/ml)	BAP (µg/l)	CTX (nmol/l)	FGF23 (ng/ml)
1	2.50	0,63	35	107	25	97	25	NA	4	NA
2	2.25	0,55	39	146	Normal	66	32	NA	10	NA
3	Normal	0,44	23	83	37	65	NA	NA	NA	NA
4	2.31	1,04	24	NA	26	83	NA	NA	NA	NA
5	2.24	0,42	26	91	29	72	15	NA	3	43
6	Normal	1,06	21	79	16	63	NA	16	Normal	NA
7	2.49	0,63	14	Normal	Undetectable	72	NA	NA	NA	NA
8	2.19	0,58	26	78	35	90	26	NA	4	NA
9	2.32	0,33	46	72	41	73	19	NA	4	60
10	2.57	2,00	16	85	Undetectable	119	54	28	NA	NA
11	2.32	0,67	34	118	51	100	18	NA	3	NA
12	2.30	0,44	35	121	19	96	19	NA	3	12
13	2.34	0,79	28	37	14	66	21	NA	4	NA
14	2.28	0,60	12	40	32	96	19	NA	2	NA
15	2.28	0,12	24	114	41	90	11	NA	3	NA
16	2.41	0,40	26	52	9	68	NA	NA	NA	NA
17	2.29	0,38	22	114	48	101	23	NA	6	41
18	2.37	0,23	19	NA	27	55	NA	NA	NA	NA
19	2.31	0,24	39	106	38	97	30	NA	4	NA
20	2.39	0,22	42	34	29	83	15	NA	1	38

^a^Unlike the other patients, the lowering of the TmPO4/GFR was inconsistent. *TmPO4/GFR* ratio of maximum rate of renal tubular reabsorption of phosphate to glomerular filtration rate, *TRP* tubular reabsorption of phosphate. Normal values: 1.3–1.85 mmol/l in adolescents, 0.85–1.5 mmol/l in adults for Serum phosphate; ≥ 0.7 mmol/l GFR for TmPO4/GFR; ≥ 80% for TRP.

*Uca* urinary calcium, *Ucr* urinary creatinine, *25OHD* calcidiol, *1,25(OH)2D* calcitriol, *PTH* parathyroid hormone, *FGF23* fibroblast growth factor 23, *MDRD* modification of diet in renal disease, *eGFR* estimated glomerular filtration rate, *OCN* Osteocalcin, *BAP* Bone-specific alkaline phosphatase, *CTX* C-terminal telopeptide of type I collagen, *FGF23* Fibroblast Growth Factor 23. Normal values: 2.25–2.6 mmol/l for Serum calcium; < 0.55 mmol/mmol Fasting for Uca/Ucr; 30–80 ng/ml for 25OHD; 20–120 pg/ml in adolescents, 15–60 pg/ml in adults for 1,25(OH)2D; 10–65 pg/ml for PTH; ≥ 60 ml/min/1.73 m^2^ for MDRD eGFR; 16–90 ng/ml in tanner 5 adolescent females, 26.6–51 ng/ml in 20–30 y/o females, 14.4–31.7 ng/ml in > 30 y/o females, 27.9–60 ng/ml in 20–30 y/o males, 21.6–38.3 ng/ml in 30–55 y/o males, 15–35.6 ng/ml in > 55 y/o males for OCN; 2.9–14.5 µg/l in premenopausal females for BAP; 0.96–4.8 nmol/l in 20–35 y/o females, 0.87–3 nmol/l in > 35 y/o females, 0.65–5.26 nmol/l in males for CTX; 23–95 ng/ml for FGF23.

**Table 2:** Genotypic characteristics of patients with SLC34A3 variants.

Various	Genetics					
Patient Id	*SLC34A3* variants (nucleotide)	*SLC34A3* variants (protein)	*Zygosity*	Initial ACMG class	Final ACMG class	
1	c.241G > A/c.846G > A	p.Gly81Ser/p.Pro282Pro	Heterozygous/heterozygous	2/4	2/4	
2	c.496G > A/c.1093 + 41_1094-15del	p.Gly166Ser/p.?	Heterozygous/heterozygous	4/4	**5**/4	
3	c.496G > A	p.Gly166Ser	Heterozygous	4	**5**	
4	c.496G > A	p.Gly166Ser	Heterozygous	4	**5**	
5	c.560 + 27_561-39del/c.575C > T	p.?/p.Ser192Leu	Heterozygous/heterozygous	5/4	5/4	
6	c.575C > T	p.Ser192Leu	Homozygous	4	4	
7	c.575C > T/c.1717_1732del	p.Ser192Leu/p.Asn573Argfs*63	Heterozygous/heterozygous	4/5	4/5	
8	c.781A > G	p.Ser261Gly	Heterozygous	2	2	
9	c.947C > T	p.Thr316Met	Heterozygous	3	3	
10	c.925 + 20_926-48del/c.1046_1047delTG	p.?/pVal349Ala fs*243	Heterozygous/heterozygous	5/5	5/5	
11	c.1208T > G	p.Met403Arg	Heterozygous	3	3	
12	c.1208T > G	p.Met403Arg	Homozygous	3	3	
13	c.1208T > G/c.1571_*80del	p.Met403Arg/p.Leu524_Leu599delins13	Heterozygous/heterozygous	3/5	3/5	
14	c.1242C > G	p.Tyr414*	Heterozygous	5	5	
15	c.1361A > G	p.Asn454Ser	Heterozygous	4	**5**	
16	c.1453C > T	p.Arg485Cys	Heterozygous	4	**5**	
17	c.1453C > T	p.Arg485Cys	Heterozygous	4	**5**	
18	c.1454G > A/c.1585A > T	p.Arg485His/p.Ile529Phe	Homozygous/homozygous	4/2	4/2	
19	c.1454G > A/c.1585A > T	p.Arg485His/p.Ile529Phe	Heterozygous/heterozygous	4/2	4/2	
20	c.1496T > C	p.Leu499Pro	Heterozygous	3	**4**	

Various	Genetics					
Patient Id	*SLC34A1* variants	ACMG class	*Zygosity*	*SLC9A3R*1 variants	ACMG class	*Zygosity*
1	No variant			No variant		
2	c.272_292del, p.p.Val91_Ala97del	3 (when heterozygous)	Heterozygous	No variant		
3	No variant			No variant		
4	c.1416 + 3G > A, p.?	3	Heterozygous	c.328C > G, p.Leu110Val	4	Heterozygous
5	No variant			No variant		
6	No variant			c.328C > G, p.Leu110Val	4	Heterozygous
7	No variant			No variant		
8	c.644 + 5G > A, p.?	3	Heterozygous	No variant		
9	No variant			No variant		
10	No variant			No variant		
11	No variant			No variant		
12	No variant			No variant		
13	No variant			No variant		
14	No variant			No variant		
15	No variant			No variant		
16	No variant			No variant		
17	No variant			No variant		
18	No variant			No variant		
19	No variant			No variant		
20	No variant			No variant		

*ACMG* American College of Medical Genetics, *SLC34A3* Solute Carrier Family 34 Member 3, *SLC34A1* Solute Carrier Family 34 Member 1, *SLC9A3R1* Solute Carrier Family 9, Member 3 Regulator 1, *hom* homozygous.

Initial ACMG classification: ACMG classification before considering phosphate uptake experiments. Final ACMG classification: ACMG classification after considering phosphate uptake experiments.

Numbering is according to the cDNA sequence (GenBank: NM_003052.4 for SLC34A1, NM_080877.2 for SLC34A3 and NM_004252.3 for SLC9A3R1). The A of the ATG of the initiator methionine codon is nucleotide 1.

Initial ACMG classification: ACMG classification before taking into account phosphate uptake experiments. Final ACMG classification: ACMG classification after taking into account phosphate uptake experiments.

### Genetic studies

Pathogenic variants in *SLC34A1*, *SLC34A3* and *SLC9A3R1* have been associated with renal phosphate leak. These genes were sequenced in all the patients, the results are shown in Table 3. Nineteen different *SLC34A3* variants were identified. Ten of these variants have not been previously reported in the literature. Among the 19 *SLC34A3* variants, 13 were considered pathogenic (initial ACMG class 4 or 5) and 6 non-pathogenic (initial ACMG class 1–3). The consequences of the mutations on protein sequence or synthesis are shown in Table 3. The ACMG classification of *SLC34A1* variants is presented in Supplementary Table 1. In addition to *SLC34A1* variants, one variant in *SLC9A3R1* and three in *SLC34A1* were also detected and are presented in Table 2. The variant in the *SLC9A3R1* gene has already been reported in patients with renal phosphate leak; this variant is responsible for functional anomalies: increased generation of cyclic AMP (cAMP) in response to parathyroid hormone (PTH) leading to inhibition of renal phosphate transport^10^. In addition, the *CYP24A1* gene was analyzed for the 3 patients with PTH either low or below the limit of detection, without detection of pathogenic variants.

**Table 3:** Genetic characteristics of SLC34A3 variants.

Patient	Variant : nucleotide	Variant : amino acid	Types of mutations	Exon Intron	Protein Domain	Initial ACMG class	Final ACMG class	Pi Uptake
1	c.241G > A	p.Gly81Ser	Missense	4	TM1	2	2	Nl
2, 3, 4	c.496G > A	p.Gly166Ser	Missense	6	IC	4	**5**	0
5, 6, 7	c.575C > T	p.Ser192Leu	Missense	7	TM3	4	4	0
8	c.781A > G	p.Ser261Gly	Missense	8	EC	2	2	> 50%
9	c.947C > T	p.Thr316Met	Missense	10	EC	3	3	> 50%
10	c.1046_1047delTG	pVal349Ala fs*243	Frameshift	10	IC	5	5	0
11, 12, 13	c.1208 T > G	p.Met403Arg	Missense	11	EC	3	3	> 50%
14	c.1242C > G	p.Tyr414*	Nonsense	12	EC	5	5	0
15	c.1361A > G	p.Asn454Ser	Missense	13	TM6	4	**5**	0
16, 17	c.1453C > T	p.Arg485Cys	Missense	13	IC	4	**5**	< 30%
18, 19	c.1454G > A	p.Arg485His	Missense	13	IC	4	4	0
20	c.1496 T > C	p.Leu499Pro	Missense	13	TM7	3	**4**	< 30%
18, 19	c.1585A > T	p.Ile529Phe	Missense	13	TM8	2	2	Nl
1	c.846G > A	p.Pro282Pro	Splice	8	EC	4	4	ND
2	c.1093 + 41_1094-15del	p.?		10	intron	4	4	ND
5	c.560 + 27_561-39del	p.?		6	intron	5	5	ND
7	c.1717_1732del	p.Asn573Argfs*63	Frameshift	13	IC	5	5	ND
10	c.925 + 20_926-48del	p.?		9	intron	5	5	ND
13	c.1571_*80del	p.Leu524_Leu599delins13	In frame	13	IC	5	5	ND

*ACMG* American College of Medical Genetics, *TM* transmembrane, *EC* extracellular, *IC* intracellular, *Nl* normal, *ND* not defined.

Initial ACMG classification: ACMG classification before considering phosphate uptake experiments. Final ACMG classification: ACMG classification after considering phosphate uptake experiments.

Numbering is according to the cDNA sequence (GenBank: NM_003052.4 for SLC34A1, NM_080877.2 for SLC34A3 and NM_004252.3 for SLC9A3R1). The A of the ATG of the initiator methionine codon is nucleotide 1.

### Expression of wild-type NPT2c in HEK cells: RT-qPCR, immunofluorescence and phosphate uptake

**Figure 1:**
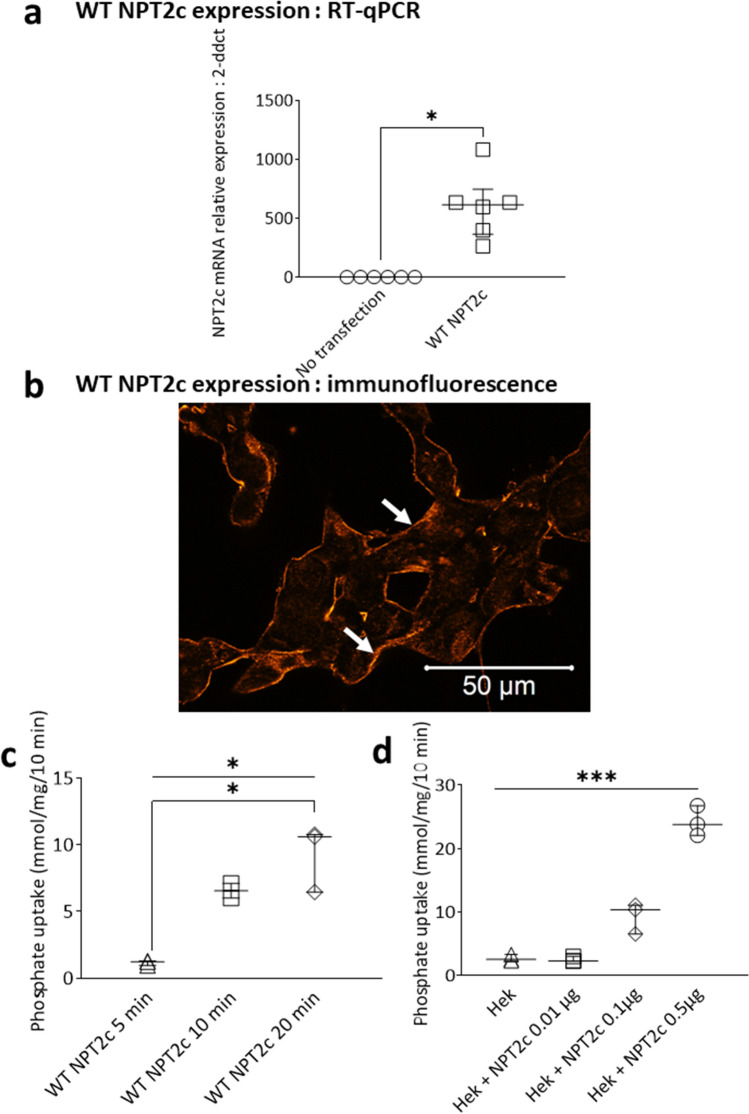
Expression of wild-type NPT2c in HEK cells. (**a**) NPT2c and GAPDH mRNA expression in HEK cells not transfected and transfected with wild-type NPT2c plasmid (0.5 μg of DNA and 1 μl of lipofectamine 2000 per well). Quantitative data represent 3 independent experiments. Statistical analysis was performed using a non-parametric Mann–Whitney test. *p < 0.05. (**b**) NPT2c immunofluorescence 48 h after transfection in HEK cells (0.5 μg of DNA and 1 μl of lipofectamine 2000 per well) localizes mainly at the plasmic membrane. (**c**) Phosphate accumulation with time in HEK cells transfected with WT NPT2c plasmid (0.5 μg of DNA and 1 μl of lipofectamine 2000 per well). Phosphate accumulation increases with time. Quantitative data represent 3 independent experiments. Statistical analysis was performed using a non-parametric Kruskall–Wallis test followed by Dunn’s multiple comparison post-hoc test. *p < 0.05. Simple line represents Kruskall–Wallis test and zig-zag lines represent Dunn’s multiple comparison post-hoc test. (**d**) Sodium-dependent phosphate uptake measured in HEK cells transfected with various amounts of WT NPT2c plasmid (0.01 μg, 0.1 µg and 0.5 µg per well). The more the amount of plasmid, the greater the phosphate uptake. Quantitative data represent 3 independent experiments. Statistical analysis was performed using a non-parametric Kruskall–Wallis test followed by Dunn’s multiple comparison post-hoc test. ***p < 0.001. Simple line represents Kruskall–Wallis test.

In order to study the consequences of the *SLC34A3* variants on NPT2c function we first characterized the properties of wild type NPT2c expressed in HEK cells, which do not naturally express NPT2c. We transfected HEK cells with NPT2c cDNA and checked by RT-qPCR experiments the expression of NPT2c mRNA (Fig. 1a). Immunofluorescence experiments confirmed that wild type NPT2c was expressed at the plasma membrane of HEK transfected cells (Fig. 1b). We then checked that wild type NPT2c membrane expression was associated with an increase in HEK cell phosphate uptake. We first studied the time dependance of phosphate uptake in HEK cells transfected with a fixed amount of NPT2c plasmid. Phosphate uptake measured until 20 min increased with time (Fig. 1c). All the subsequent uptake experiments were performed at 10 min of incubation. We transfected HEK cells with increasing amount of wild type NPT2c plasmid. Phosphate uptake increased with NPT2c cDNA amount. (Fig. 1d).

### Functional characterization of NPT2c mutants

We studied the consequences of 13 variants (11 missense, 1 nonsense and 1 frameshift) that result in a modification of the NPT2c protein sequence. We did not study the consequences of the mutations located in the introns.

**Figure 2:**
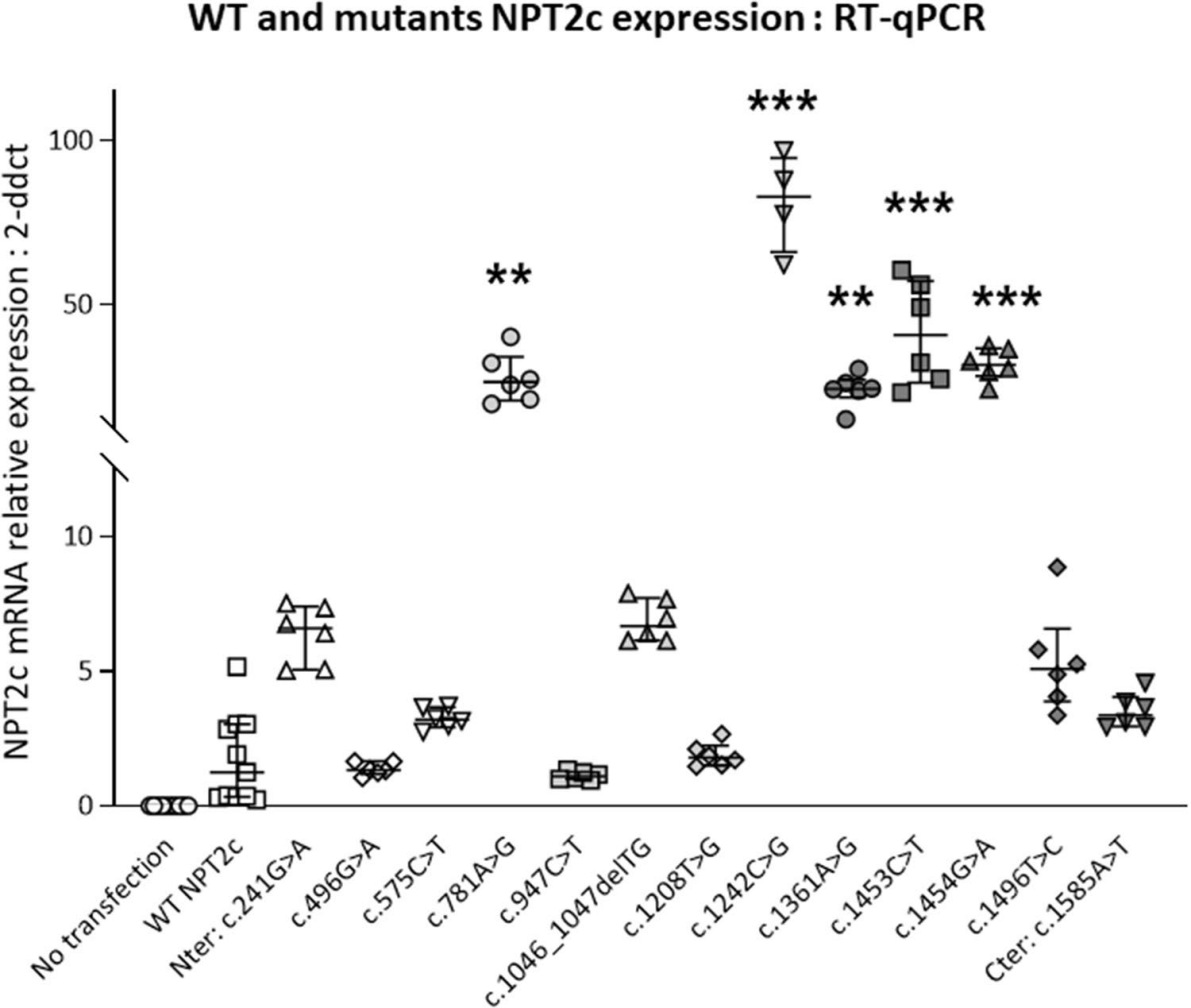
Comparison of the level of expression of NPT2c mRNA after transfection. HEK cells were transfected with a wild type or a mutant NPT2c or a control plasmid (0.5 μg of DNA and 1 μl of lipofectamine 2000 per well). NPT2c and GAPDH mRNA expression were quantified by RT-PCR. Quantitative data represent at least 4 independent experiments. Statistical analysis was performed using a non-parametric Kruskall–Wallis test followed by Dunn’s multiple comparison post-hoc test. *p < 0.05, **p < 0.01, ***p < 0.001, ****p < 0.0001. Simple line represents Kruskall–Wallis test and zig-zag lines represent Dunn’s multiple comparison post-hoc test.

**Figure 3:**
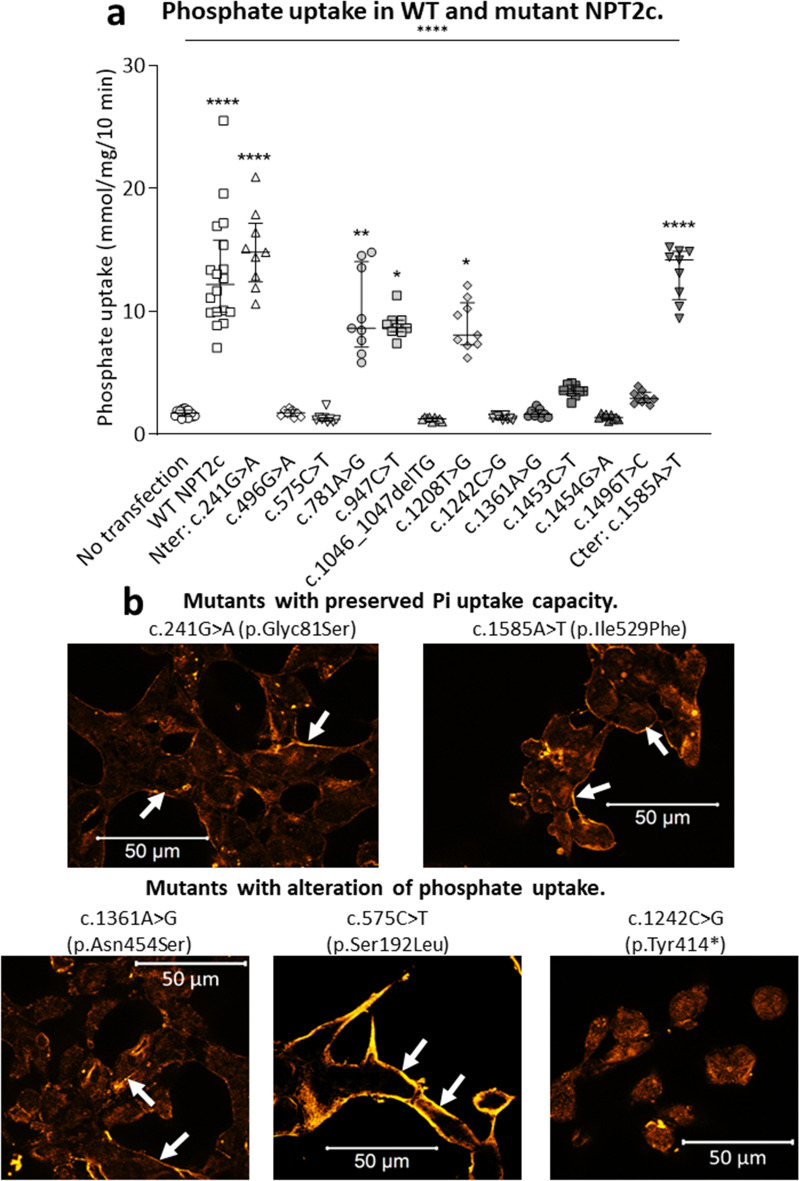
In vitro characterization of the NPT2c variants. (**a**) Na-dependent phosphate uptake: HEK expressing WT NPT2c or NPT2c variants (cells transfected with 0.5 μg of DNA and 1 μl of lipofectamine 2000 per well). 1 variant transport phosphate normally, 4 variants have a phosphate transport function slightly lowered et 8 have a strong decrease in phosphate transport. Quantitative data represent at least 9 independent experiments. Statistical analysis was performed using a non-parametric Kruskall–Wallis test followed by Dunn’s multiple comparison post-hoc test. ****p < 0.0001, simple line represents Kruskall–Wallis test. *p < 0.05, **p < 0.01, ****p < 0.0001 on NPT2c conditions represent Dunn’s multiple comparison post-hoc test compared to non- transfected cells. (**b**) We performed immunofluorescence on HEK cells transfected with transporting and non-transporting NPT2c variants (cells transfected with 0.5 μg of DNA and 1 μl of lipofectamine 2000 per well). All the transporting variants were correctly addressed at the plasmic membrane. Some of the non-transporting variants were not addressed at the plasmic membrane (e.g. c.1242C > G, p.Tyr414*), others were correctly addressed at the plasmic membrane (e.g. c.575C > T, p.Ser192Leu and c.1361A > G, p.Asn454Ser).

The NPT2c variants were inserted by site-directed mutagenesis and included in expression vectors. All the cDNA were sequenced to verify the presence of the variant and the absence of additional modification in the sequence. We then transiently expressed the cDNAs of these mutants in HEK cells. We checked NPT2c RNA expression in transfected HEK cells by rt-QPCR (Fig. 2). NPT2c mRNA was detected in all transfected cells but was undetectable in non-transfected HEK cells. We assessed the functionality of the NPT2c mutants by measuring phosphate uptake in the transfected HEK cells (Fig. 3a). Phosphate uptake of HEK transfected cells was compared to sham-transfected cells. Phosphate transport was significantly affected in some but not all NPT2c mutants. Mutants c.241G > A (p.Gly81Ser) and c.1585A > T (p.Ile529Phe) exhibited phosphate transport capacity similar to wild type NPT2c (Fig. 3a). Phosphate uptake was moderately diminished (24–32%) in HEK cells transfected with mutants c.781A > G (p.Ser261Gly), c.947C > T (p.Thr316Met) or c.1208T > G (p.Met403Arg). Phosphate accumulation was almost completely abolished in 8 mutants: c.496G > A (p.Glyc166Ser), c.575C > T (p.Ser192Leu), c.1046_1047delTG (p.Val349Alafs*243), c.1242C > G (p.Tyr414*), c.1361A > G (p.Asn454Ser), c.1453C > T (p.Arg485Cys), c.1454G > A (p.Arg485His) and c.1496 T > C (p.Leu499Pro) (Fig. 3a). Based on these results, we reclassified the ACMG class of each patient as the final ACMG class (Tables 2, 3). The ACMG class was modified for 4 of the variants. We analyzed by immunofluorescence the expression of the NPT2c proteins in transfected HEK cells (Fig. 3b). All the mutants that retained the ability to transport phosphate was expressed at the cell surface. Among the mutants that did not transport phosphate some were properly addressed at the cell surface, others were trapped in the cytoplasm.

### Effect of the co-expression of wild-type NPT2a or wild-type NPT2c with NPT2c mutants

**Figure 4:**
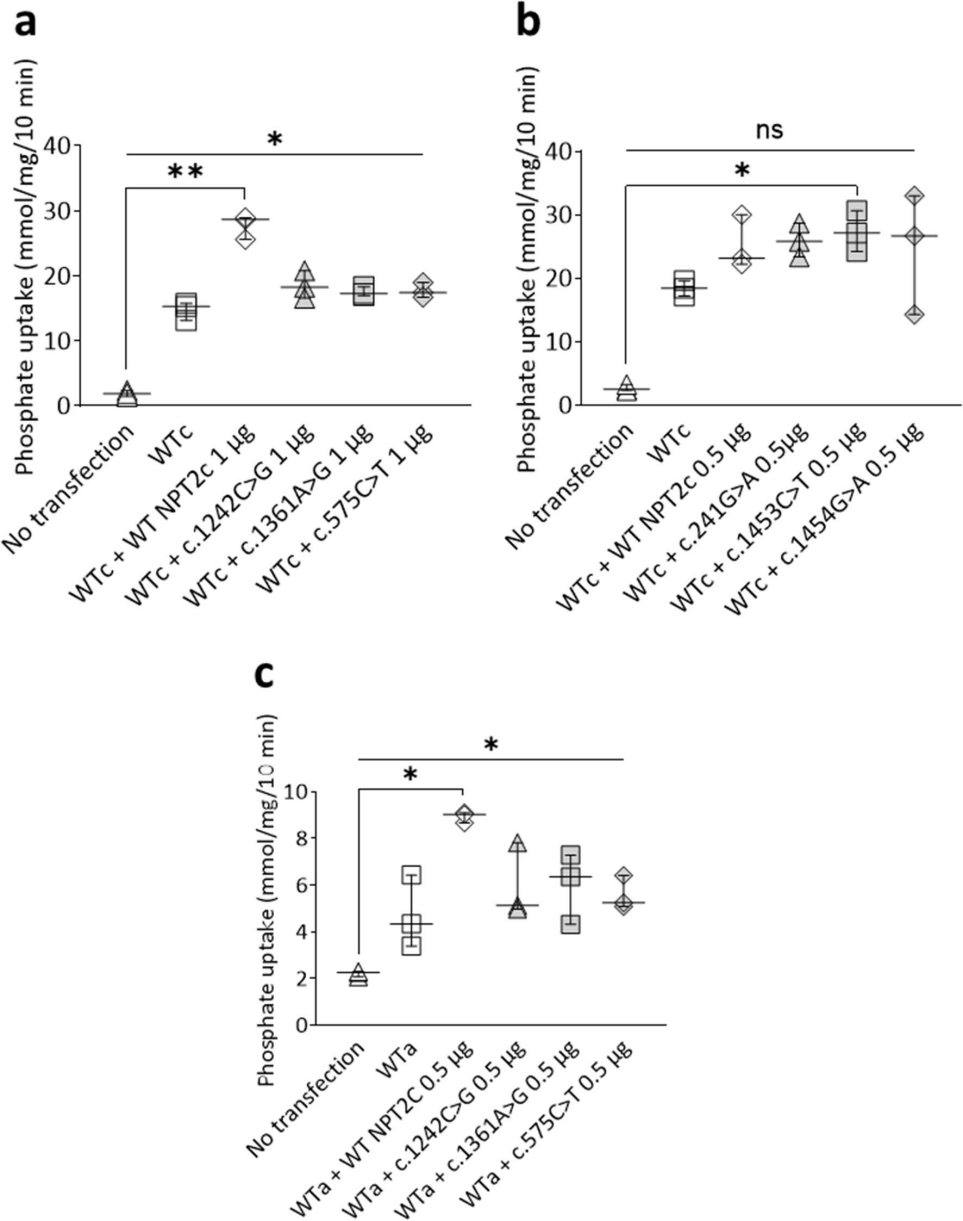
NPT2c variants do not inhibit either WT NPT2c or WT NPT2a phosphate transport activity. (**a**) Na-dependent Phosphate uptake comparison between non transfected HEK cells (No transfection), HEK cells stably transfected with WT NPT2c (WT) and HEK cells transfected stably with WT NPT2c and transiently transfected with either WT NPT2c (WT + WT NPT2c) or non-transporting NPT2c variants (WT + variant). Transiently transfected cells with 1 μg of DNA and 1 μl of lipofectamine 2000 per well. Expression of non-transporting NPT2c variants (WT + variant) did not decrease sodium phosphate transport compared to the HEK cells expressing WT NPT2c alone (WT). Quantitative data represent 3 independent experiments. Statistical analysis was performed using a non-parametric Kruskall–Wallis test followed by Dunn’s multiple comparison post-hoc test. *p < 0.05, **p < 0.01. Simple line represents Kruskall–Wallis test and zig-zag line represent Dunn’s multiple comparison post-hoc test. (**b**) Similar experiment with variants that do transport phosphate. With the HEK cells transfected stably with WT NPT2c and transiently with NPT2c variants that do transport phosphate (WT + variant), phosphate uptake is similar to HEK cells transfected stably and transiently with WT NPT2c (WT + WT NPT2c) and higher than WT NPT2c alone (WT). So, expression of the transporting NPT2c variants did not decrease phosphate transport compared to the HEK cells expressing WT NPT2c. Transiently transfected cells with 0.5 μg of DNA and 1 μl of lipofectamine 2000 per well. Quantitative data represent 3 independent experiments. Statistical analysis was performed using a non-parametric Kruskall–Wallis test followed by Dunn’s multiple comparison post-hoc test. *p < 0.05, ^ns^p not significant. Simple line represents Kruskall–Wallis test and zig-zag line represent Dunn’s multiple comparison post-hoc test. (**c**) Similarly, we checked whether non transporting NPT2c variants could decrease phosphate transport in HEK cells expressing WT NPT2a. Again, transient expression of the non-transporting NPT2C variants in HEK Cells stably transfected with WT NPT2a (WT + variant) did not decrease phosphate transport compared to the HEK cells expressing stably WT NPT2a (WT). Transiently transfected cells with 0.5 μg of DNA and 1 μl of lipofectamine 2000 per well. Quantitative data represent 3 independent experiments. Statistical analysis was performed using a non-parametric Kruskall–Wallis test followed by Dunn’s multiple comparison post-hoc test. *p < 0.05. Simple line represents Kruskall–Wallis test and zig-zag line represent Dunn’s multiple comparison post-hoc test.

To find an explanation to the existence of urinary phosphate loss in patients with a NPT2c mutant that moderately reduces phosphate uptake in vitro we checked if these mutants could interfere with wild type NPT2c or wild type NPT2a functions. Co-transfection of various cDNA amounts of three NPT2c mutants that did not transport phosphate with wild-type NPT2c cDNA did not decrease wild type NPT2c mediated phosphate uptake (Fig. 4a). Co-transfection of NPT2c mutants transporting phosphate with wild-type NPT2c led to increased phosphate transport equivalent to wild-type NPT2c double transfection (Fig. 4b). In conclusion phosphate transport capacity of wild-type NPT2c was not altered by NPT2c mutants. We similarly check if NPT2c mutants could diminish NPT2a mediated phosphate transport. None of the NPT2c mutants significantly altered NPT2a mediated phosphate uptake (Fig. 4c). All these experiments show that there is no negative dominant effect of NPT2c mutants on wild-type NPT2c or NPT2a.

## Discussion

We describe here a group of patients with recurrent nephrolithiasis for whom genetic exploration revealed variants in the *SLC34A3* gene that codes for NPT2c protein.

We analyzed the phenotype of these patients through precise renal and mineral metabolism exploration. All patients had low TmP/GFR or TRP, in one patient the decrease in TmP/GFR was fluctuating. Plasma concentrations of PTH were normal except for 3 patients (PTH either low or undetectable) and FGF23 was normal when measured. We expressed in HEK cells 13 of the 19 NPT2c mutants found in our patients. We did not express 6 variants among which 3 cause large intronic deletions very likely promoting splicing alteration. Five of these 6 variants have already been reported: c.846G > A^11^, c.1093 + 41_1094-15del^12^, c.560 + 27_561-39del^13^, c.925 + 20_926-48del^14^, c.1571_*80del^13^. Variant c.1717_1732del (p.Asn573Argfs*63) creates a frameshift that markedly modify the protein sequence and to our knowledge has not been reported before.

We studied in vitro phosphate transport capacity of 13 mutants. Four have already been reported in the literature, but only one (p.Ser192Leu) had its function characterized. This mutant was expressed in HEK cells and Xenopus oocytes by Schönauer and coll^15^. As reported by Schönauer and coll. we found that p.Ser192Leu did not transport phosphate although it was partially addressed at the plasma membrane. Mutants pVal349Ala fs*243, p.Arg485His and p.Ile529Phe have already been observed, however the authors did not perform functional studies^13,16^. Our data show that phosphate uptake was markedly diminished by mutants pVal349Ala fs*243 and p.Arg485His but was unaltered by mutant p.Ile529Phe.

For all patients we looked for variants in the genes encoding NHERF1 or NPT2a. Indeed, pathogenic variants in these genes can cause urinary phosphate loss and urolithiasis. Three patients had variants in the SLC34A1 gene (#2, #4 and #8) and two had a pathogenic variant in the SLC9A3R1 gene (#4 and #6).

In patient #8, the variant c.644 + 5G > A in *SLC34A1* gene that encodes NPT2a is located in the splice donor site of intron 6, in silico analysis predicted that this may affect a splicing site. According to ACMG classification criteria this variant is class 3 (uncertain significance). The consequences on NPT2a protein are unknown and have not been reported so far. However, a similar mutation involving the last nucleotide of the sixth exon of SLC34A1 gene has been reported (c.644 + 1G > A)^17,18^. This mutation is associated with an idiopathic infantile hypercalcemia phenotype in the homozygous state and with hypercalciuria and nephrocalcinosis in the heterozygous state. Unfortunately, no sample was available to study the consequences of the transcript. Patient #8 also had a variant in the *SLC34A3* gene (c.781A > G) that reduces NPT2c-mediated phosphate uptake by only 24% making unlikely that this mutation alone can explain the low TmP/GFR of this patient. Our observations suggest that the low TmPO4/GFR value was probably mainly explained by the NPT2a mutation.

The *SLC34A1* variant c.272_292del, identified in patient #2 has already been reported by different authors^17,19^ with unclear consequences on phosphate transport: phosphate uptake was found either normal^17^ or decreased^19^ in cells expressing this mutant. In our study patient #2 who had this variant also presented two variants in the *SLC34A3* gene that alter phosphate transport, consequently the contribution of the *SLC34A1* variant to the patient phenotype is unlikely.

The *SLC34A1* variant c.1416 + 3G > A found in patient #4 has never been reported to our knowledge, however, a similar mutation (c.1416 + 5G > A) has been observed^17,20^. These mutations modify a splicing site, with predicted deleterious effect on NPT2a function. We cannot analyze the consequences on the transcript of the c.1416 + 3G > A variant. In addition, patient #4 also presented a mutation in NHERF1 gene known to alter phosphate transport control and a mutation in the NPT2c gene that markedly reduces phosphate uptake, which is likely responsible for his phenotype.

Patients #4 and #6 presented the same *SLC9A3R1* variant: c.328C > G (p.Leu110Val). This mutant has already been characterized and reported as pathogenic by Karim and coll^10^.

All NPT2c mutants that were classified 4 or 5 according to the criteria of the ACMG showed a strong decrease in their capacity to transport phosphate. On the other hand, all mutants categorized 2 according to the ACMG criteria showed normal or mild decreases in phosphate uptake. By contrast, our study has enabled the reclassification of some uncertain signification variants (class 3 ACMG). Four NPT2c mutants were categorized ACMG 3: p.Gly81Ser, p.Thr316Met, p.Met403Arg, and p.Leu499Pro. p.Gly81Ser mutant transported phosphate normally. Under our experimental conditions (0.1 mM of phosphate concentration) we would have detected a significant decrease in the affinity of the transporter for phosphate (increase of the Km). Mutants p.Thr316Met (c.947C > T) and p.Met403Arg (c.1208T > G) retained a substantial capacity to transport phosphate. Mutant p.Leu499Pro (c.1496T > C) abolished almost completely phosphate uptake. These data show the importance of functional studies on phosphate uptake to go further with the interpretation of ACGM class 3 variants.

We have analyzed the relationship between the patient's phenotypes and the mutations identified in *SLC34A3* gene.

The *SLC34A3* variant c.496G > A, detected in patients #2 (associated in another SLC34A3 heterozygous variant), #3, and #4 (as heterozygous), almost completely abolished phosphate uptake. Patient #4 has in addition a *SLC34A1* class 3 variant and a *SLC9A3R1* class 4 variant. Patients #3, and #4 are two male subjects who had almost the same age, but Patient #4 presented a slightly more severe phenotype compared to Patient #3 with a shorter height, lower serum phosphate concentration, despite lower serum PTH concentration, and higher urinary calcium excretion. These differences may be accounted for by the mutation of *SLC9A3R1* that is known to induce urinary phosphate loss^10^; the consequences of the mutation in the *SLC34A1* gene being less well characterized.

Patient #6 is homozygous for *SLC34A3* variant c.575 > T. This variant was also present in patient #5 and #7 associated with a second pathogenic variant of the same gene. Comparison of the phenotype of these 3 patients is made difficult by the differences in age at the time of the diagnosis. Estimated glomerular filtration rate is lower in Patient #6 and urinary calcium excretion higher than in Patients #5 and #7; however we cannot conclude if the association of this homozygous variant with the *SLC9A3R1* class 4 variant is responsible for a more severe phenotype.

Three *SLC34A3* variants that deeply lessen phosphate transport were found in several unrelated patients (c.496G > A, c.575C > T, c.1453C > T).

Patient #3 was heterozygous for mutation c.496G > A and had no mutations in NPT2a or NHERF1 genes by contrast with patients #2 and #4. Serum phosphate concentration and urinary calcium excretion were almost normal in patient #3. Serum phosphate concentration was higher and urinary calcium excretion was lower than in patient #2 and Patient #4, suggesting a less severe phenotype.

Variant c.575C > T, that completely abolishes phosphate uptake was found in three patients: associated with a second *SLC34A3* pathogenic variant (patients #5 and #7), or homozygous (patient #6). In addition, this last patient also harbors a likely pathogenic *SLC9A3R1* variant. These observations suggest that when a mutation has been identified in one candidate gene the analysis of other candidate genes is necessary.

Patients #16 and #17 were heterozygous for variant c.1453C > T with no other variants identified suggesting it could explain the diminution of serum phosphate concentration.

Variant c.1208T > G was present in 3 patients: patient #11 was heterozygous, patient #12 was homozygous and patient #13 was likely compound heterozygous. This mutant only slightly decreased NPT2c-mediated phosphate uptake. Surprisingly the phenotype of patients #12 and #13, regarding serum phosphate concentrations and TmPO4/GFR values, was not more severe than that of patient #11. These findings question the responsibility of this variant in the phenotype of these patients and this variant could correspond to a rare polymorphism with no pathological significance. The allelic frequency of this variant has been reported at 6.26^–5^^21^.

Similarly, patient #9 was heterozygous for a mutation in SLC34A3 gene (c.947C > T) that does not markedly alter NPT2c phosphate transport capacity suggesting that this variant is a polymorphism.

Patients #1 has a *SLC34A3* non-pathogenic variant (c.241G > A, normal in vitro phosphate uptake). This variant is probably a polymorphism. This patient harbors another *SLC34A3*variant previously described by Lorenz-Depiereux and coll^11^. This mutant, c.846G > A does not modify protein sequence (p.Pro282Pro), but might lead to an aberrant splicing product although this has not been proven by checking mRNA expression. However, the Human Splicing Finders Software^22^ support the idea that this mutation leads to modified splicing. Two patients harbor two variants (p.Arg485His and p.Ile529Phe): patient #18 is homozygous for these variants and patient #19 is heterozygous. We have detected this same association in additional patients analyzed by next generation sequencing; in these additional cases these two variants are in the same allele. DNA samples of relatives were not available to establish whether these variants are also *in cis* in patients #18 and #19. We tested each mutation individually; one abolished phosphate transport (p.Arg485His) the other (p.Ile529Phe) did not alter phosphate transport. Although we did not introduce these two mutations in the same cDNA, we can anticipate that the combination of these two mutations on the same DNA strand would result in a decrease in phosphate uptake. Arg485 seems to be a sensitive amino acid for NPT2c phosphate transport capacity, since two mutations replacing this amino acid, identified and studied here, p.Arg485His and p.Arg485Cys, showed severely hampered phosphate uptake.

We speculated that NPT2c mutant protein might interfere with wild type protein resulting in a decrease in phosphate transport. Our experiments did not observe an inhibitory effect of the NPT2c mutants on phosphate uptake mediated by wild type NPT2c or NPT2a.

In our study, the analysis of the relationship between genotype and phenotype of patients was limited by the absence of segregation analysis. This analysis could not be conducted because of the difficulty to obtain genetic samples from relatives.

## In conclusion

Finding of variants in the genes *SLC34A1*, *SLC34A3* and *SLC9A3R1* is not uncommon in patients with renal phosphate leak and kidney stones. The presence of variants in more than one of these genes in the same patient is not an unusual finding, consequently these three genes should be systematically sequenced in subjects suspect of hereditary phosphate loss of renal origin.

The identification of a mutant in NPT2c should be associated with a functional study of the protein since phosphate uptake capacity may not be affected, in mutants classified as uncertain significance (ACMG class 3) in particular.

## Materials and methods

### Patients

We analyzed the phenotypes of patients initially referred to our institution for recurrent renal lithiasis and with at least one relative with recurrent renal stones. Patients had a complete exploration of the phosphate and calcium metabolism in the fasting state after one day of a calcium free diet, including a mineral water without calcium. In all these patients 3 genes associated with renal phosphate leak: *SLC34A1* (encoding NPT2a), *SLC34A3* (encoding NPT2c) and *SLC9A3R1* (encoding NHERF1) were sequenced in the department of genetics of Hôpital Européen Georges Pompidou.

### *SLC34A3* genetic analyses

Informed consents for genetic study were obtained and genetic testing was performed in accordance with French legislation on genetics diagnostics tests (French bioethics law no. 2004-800).

Total DNA was extracted from blood peripheral leucocytes by standard procedures. Mutation analysis was performed by PCR amplification and direct sequencing of exons, flanking intronic sequences and complete short introns (2, 4, 6, 8, 9 and 10) of the *SLC34A3* gene, with specific primers (conditions on request) and then sequenced using BigDye Terminator kit v3.1 cycle sequencing kits and run on an ABI Prism 3730XL DNA Analyzer (Life Technologies, Foster City, CA).

DNA mutants were identified using Sequencer software (Gene Codes Corporation, Ann Arbor, MI) by comparison with the reference sequence for SLC34A3: NM_080877.2. Each mutation was confirmed by sequencing a second independent PCR product.

Mutants were interpreted using Alamut Visual 2.10 software (Interactive Biosoftware; Rouen France, http://www.interactivebiosoftware.com). Allelic frequencies were determined using the gnomAD database (https://gnomad.broadinstitute.org/). Mutants were classified according to the American College of Molecular Genetics guidelines^23^.

### Wild-type NPT2c and NPT2c mutants and NPT2a plasmids

We acquired the NPT2c (ref : RC222058) and NPT2a (ref : RC208782) plasmids from OriGene Technologies. The NPT2c plasmid was tagged with the FLAG epitope. The 13 NPT2c mutants were generated from wild-type NPT2c plasmid using QuickChangeII Site-Directed Mutagenesis kit (Agilent Technologies). All constructs were sequenced to verify introduction of the correct mutants and the absence of cloning artifacts.

### Cell culture conditions and transfections

For maintenance, HEK cells were seeded at 1700 cells/cm^2^, and medium was renewed once a week. HEK cells were maintained in DMEM/nutrient mixture F12 supplemented with 2.5 mM glutamine, 15 mM HEPES, and 9.1% FBS. For transient plasmid transfection, HEK cells were seeded 48 h before the experiment in antibiotic-free medium at 160,000 cells per well in a 24-well plate. HEK cells were transfected with plasmid using Lipofectamine 2000 (Invitrogen) in 500 µl of a serum free medium according to the manufacturer’s instructions. Six hours after transfection, 5% FBS was added to the medium. Other HEK cells were stably transfected with wild-type NPT2c plasmid and maintained in selection media (geneticin).

### Gene expression analysis

Total RNA was isolated from cells using NucleoSpin RNA II columns (Macherey Nagel). RT-PCR amplifications were performed using M-MLV (Invitrogen) according to the manufacturer’s instructions. Realtime PCR was performed using SYBR Green chemistry (ThermoFisher Scientific) on an ABI Prism 7000 detection system. The GAPDH gene was used as the reference gene^24^. Oligonucleotide primers were as follows: SLC34A3 forward 5′-CATCATCATGGGTGTCAACGTAGG-3′, *SLC34A3* reverse 5′-GCTGAAAGCCCTCTGAAATTCATC-3′; *GAPDH* forward 5′-GGCTCTCCAGAACATCATCCCTGC-3′, *GAPDH* reverse 5′-GGGTGTCGCTGTTGAAGTCAGAGG-3′. Primer specificity was assessed by multiple dilution and melt curve analysis. The specificity of the primers was evaluated by dilution range of a control sample cDNA and by analysis of the melting curves.

### Phosphate uptake experiments

Radiolabeled phosphate uptake was measured as previously described^10^ by incubating cells for 10 min in iso-osmotic transport medium containing or not Na and 0.5 µCi/ml ^32^P and 0.1 mM of Pi (KH_2_PO_4_). Cells were washed 3 times with 137 mM NaCl cold medium (pH 7,4) and solubilized with 100 mM NaOH. The radioactivity of the lysate was counted by liquid scintillation spectroscopy. Each assay was performed in triplicate and total protein content was measured by the Bradford assay (ThermoFischer Scientific). The amount of phosphate absorbed by the cells was expressed in nmol of phosphate divided by the amount of protein per condition. Depending on the conditions, FGF23 was incubated for 20 min before the experiment at 6 ng/ml.

### Immunofluorescence microscopy analysis

HEK cells grown on 8 well µ-Slide (Ibidi, 80826) were fixed with 4% paraformaldehyde for 20 min at room temperature, incubated in blocking solution (10% fetal bovine serum in PBS) for 30 min. And cells were then incubated with either an anti-FLAG epitope primary antibody or an anti Na,K-ATPase alpha 1 subunit primary antibody in permeabilization/blocking buffer (PBS with 10% fetal bovine serum and 0.05% saponin from Sigma Aldrich, 8047) for 1 h at room temperature. Subsequently, cells were incubated with Alexa Fluor 548 nm secondary antibody for 1 h at room temperature. Coverslips were mounted using DAPI Fluoromount-G (Southern biotech) to visualize nuclei. Images were obtained using a Zeiss Apotome.2 fluorescence microscope using a 63 × oil immersion objective. The images were analyzed with ZEN software (Carl Zeiss Microscopy GmbH).

### Statistical analysis and reproducibility

The quantitative data are represented by scatter plots. The lines represent the mean and the error bars the standard deviation. For the real-time PCR and phosphate transport experiments, differences between groups were analyzed by the non-parametric Kruskall–Wallis test followed by multiple comparisons of Dunn's post-hoc test. The significance level was defined as p ≤ 0.05. All statistical analyses were performed with Graphpad Prism (Graphpad) software.

### Ethic statements

The study protocol complies with the Declaration of Helsinki and received approval from the institutional review boards of the Assistance Publique Hôpitaux de Paris.

## Supplementary Information

Supplementary Information.

## Data Availability

The datasets generated during and/or analyzed during the current study are available from the corresponding author on reasonable request. The data regarding gene polymorphisms have been submitted to ClinVar: submission ID SUB11518361, Organization number: 508350. The accession numbers for each variant are as follow: SCV002513790; SCV002513801; SCV002513812; SCV002513823; SCV002513834; SCV002513845; SCV002513856; SCV002513867; SCV002513869; SCV002513791; SCV002513792; SCV002513793.

## Abbreviations

1,25(OH)_2_D: Calcitriol
25OHD: Calcidiol
ACMG: American college of medical genetics
eGFR: Estimated glomerular filtration rate
FGF23: Fibroblast growth factor 23
HHRH: Hereditary hypophosphatemic rickets with hypercalciuria
MDRD: Modification of diet in renal disease
NHERF1: Sodium/hydrogen exchanger regulatory factor 1
NPT2a: Sodium-dependent phosphate transporter 2a, NaPi-IIa, Npt2a
NPT2c: Sodium-dependent phosphate transporter 2c, NaPi-IIc, Npt2c
PiT1: Inorganic phosphate transporter-1
PTH: Parathyroid hormone
*SLC20A1*: Solute carrier family 20 (phosphate transporter), member 1
*SLC34A1*: Solute carrier family 34 (type 2 sodium/phosphate cotransporter), member 1
*SLC34A3*: Solute carrier family 34 (sodium/phosphate cotransporter), member 3
*SLC9A3R1*: Solute carrier family 9, member 3, regulator 1
TmPO_4_/GFR: Ratio of maximum rate of renal tubular reabsorption of phosphate to glomerular filtration rate
TRP: Tubular reabsorption of phosphate
Uca: Urinary calcium
Ucr: Urinary creatinine
VUS: Variant of uncertain significance
