# Supplementary material for: Relationship between clinical phenotype and in vitro analysis of 13 NPT2c/SCL34A3 mutants
Source: Sci Rep. 2023 Jan 3;13:85. doi: 10.1038/s41598-022-25995-5 (PMC9810644; doi:10.1038/s41598-022-25995-5)
Supplement: Supplementary file 1 — Supplementary Information. [file 41598_2022_25995_MOESM1_ESM.pdf]

a

Supplemental figure S-1, Panel a

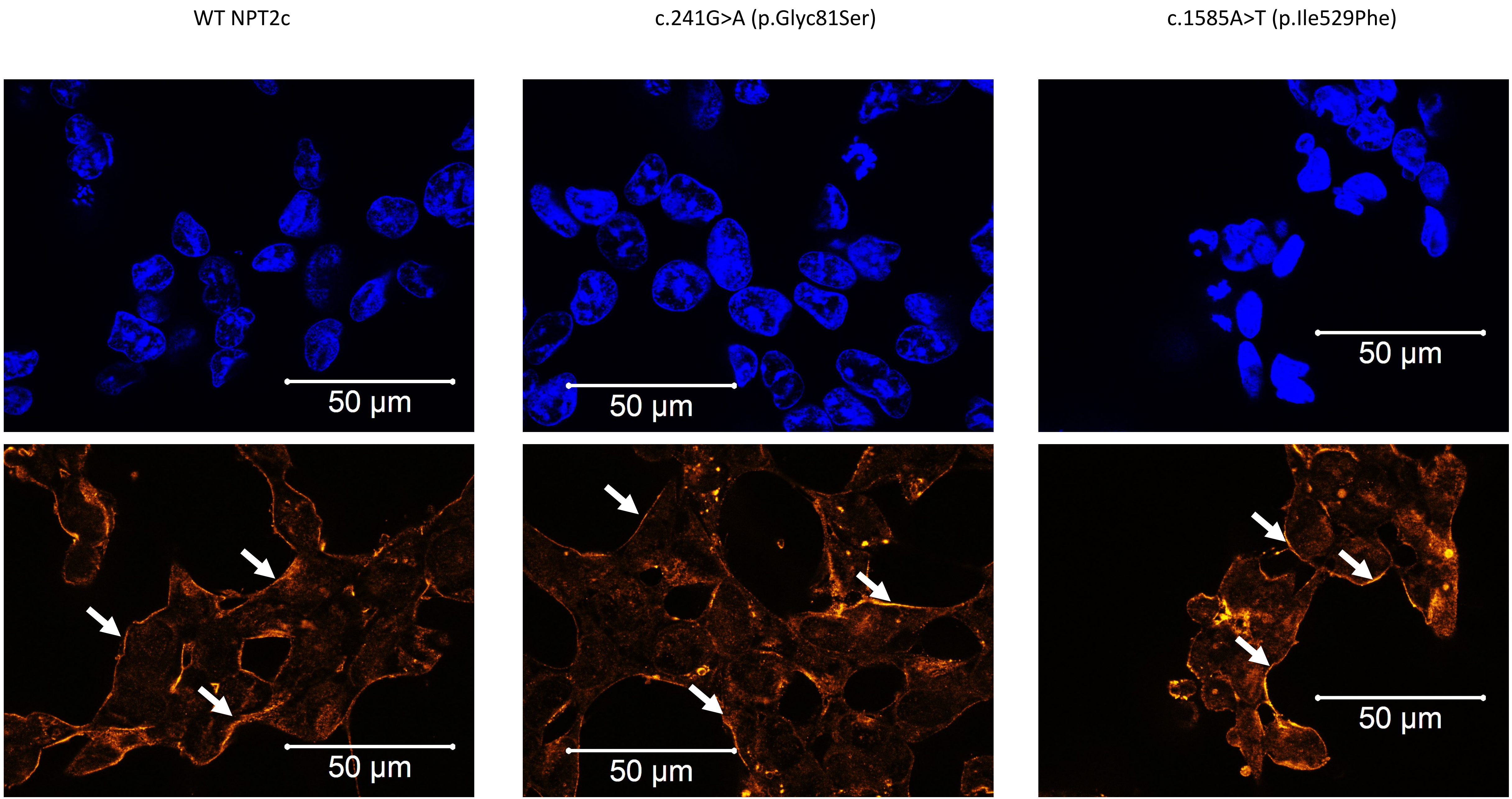

Supplemental figure S-1, Panel a, continues

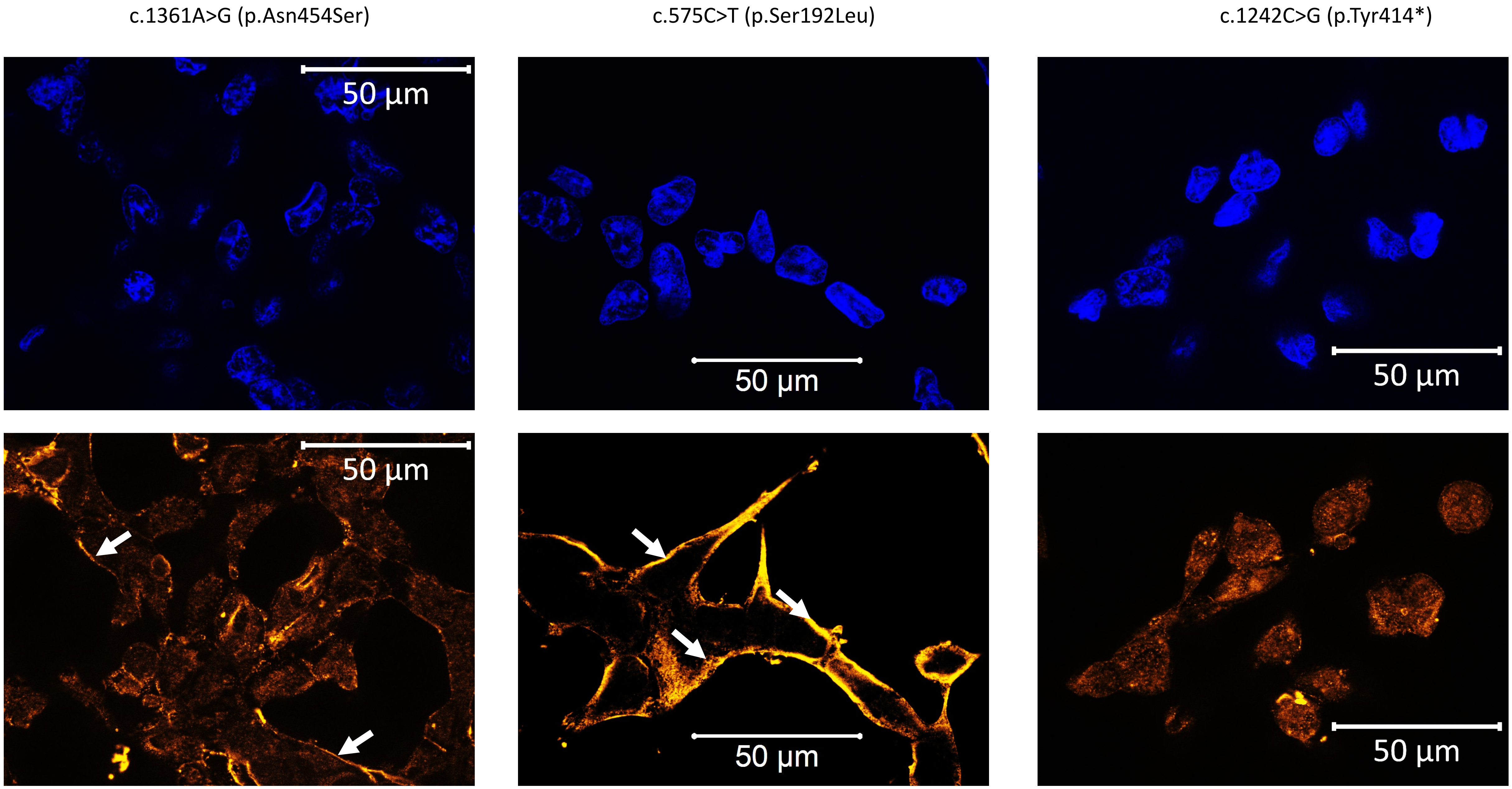

b

Supplemental figure S-1, Panel b

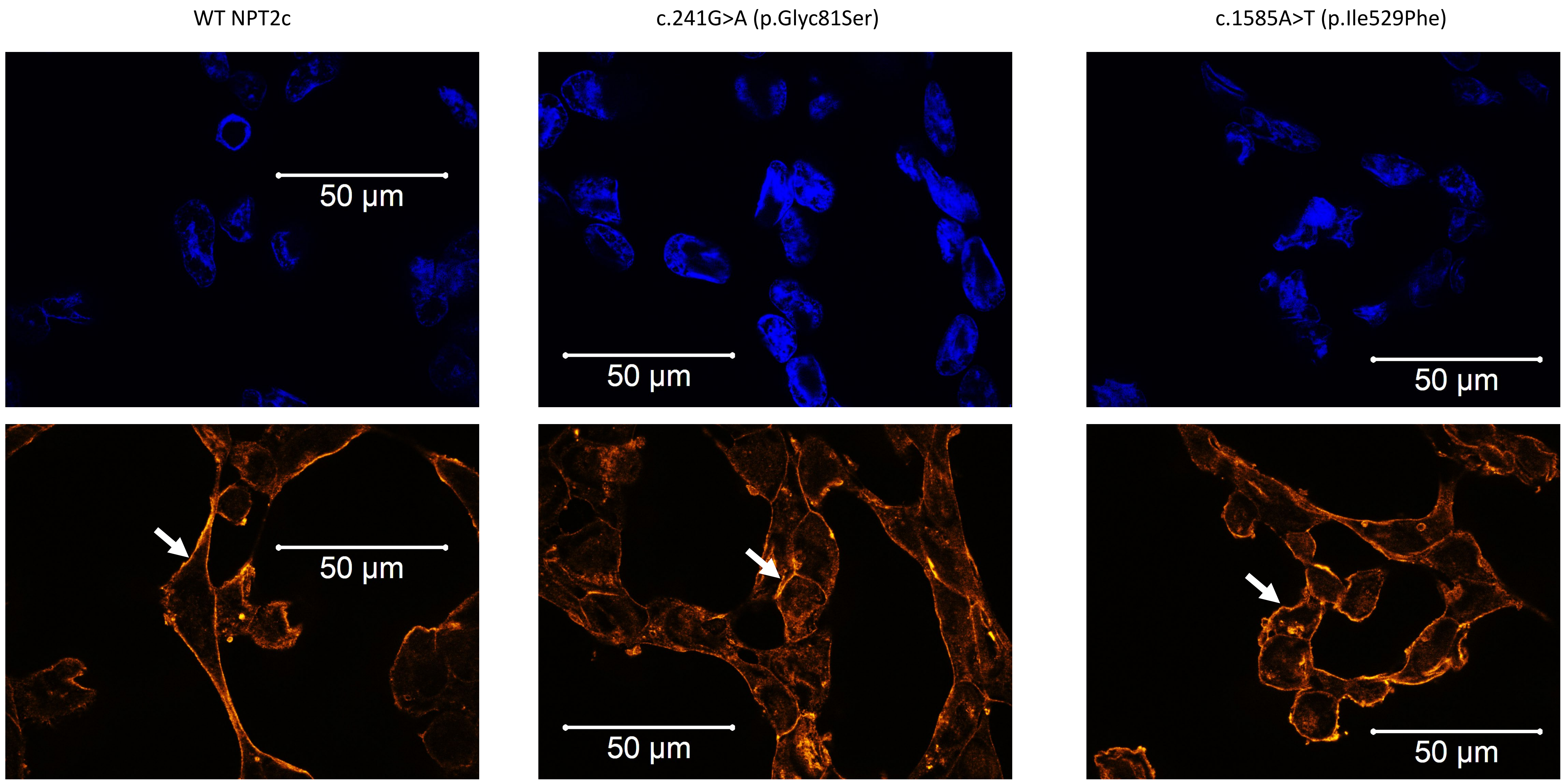

Supplemental figure S-1, Panel b, continues

c.1361A>G (p.Asn454Ser) c.575C>T (p.Ser192Leu) c.1242C>G (p.Tyr414*)

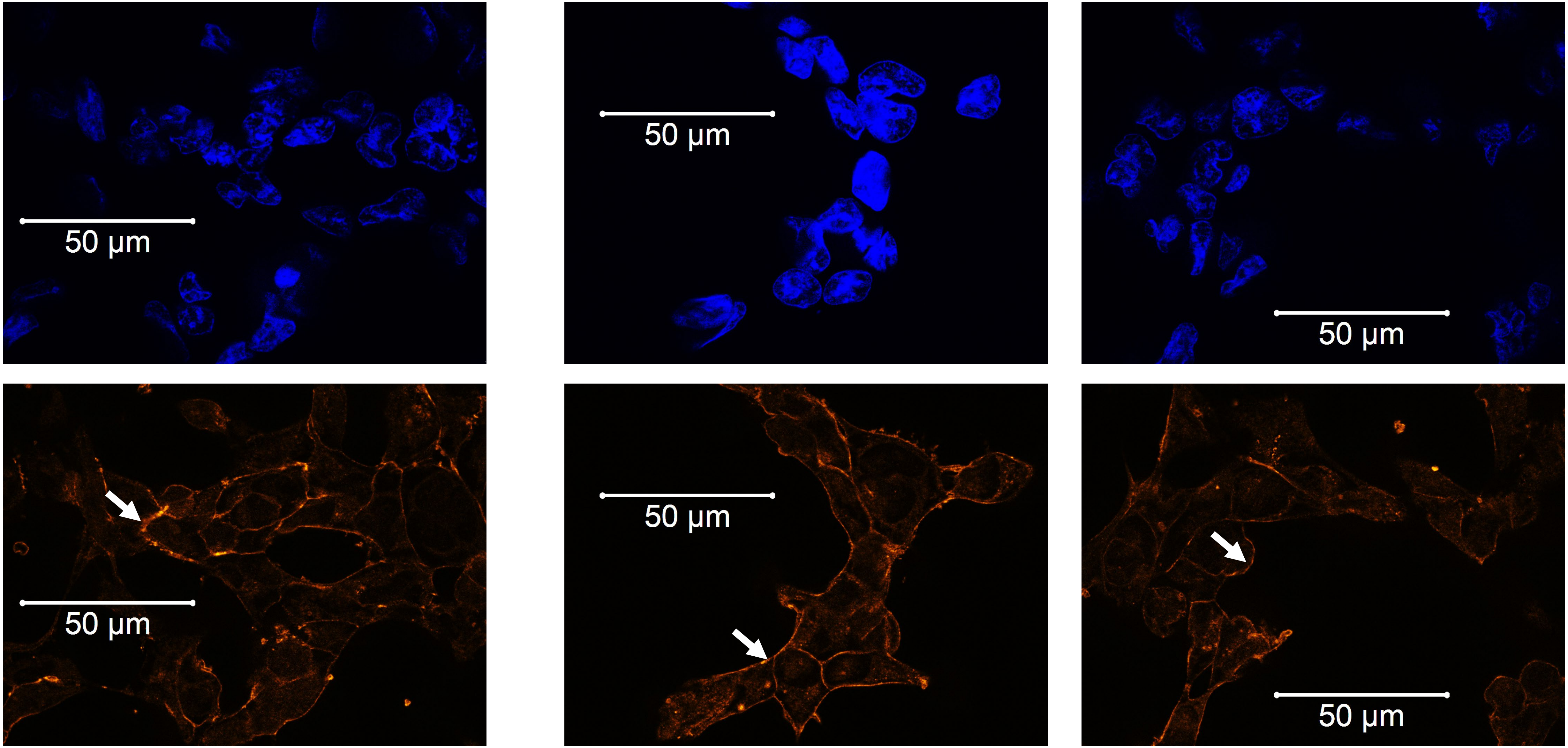

Supplemental figure S-1, Panel c

WT NPT2c

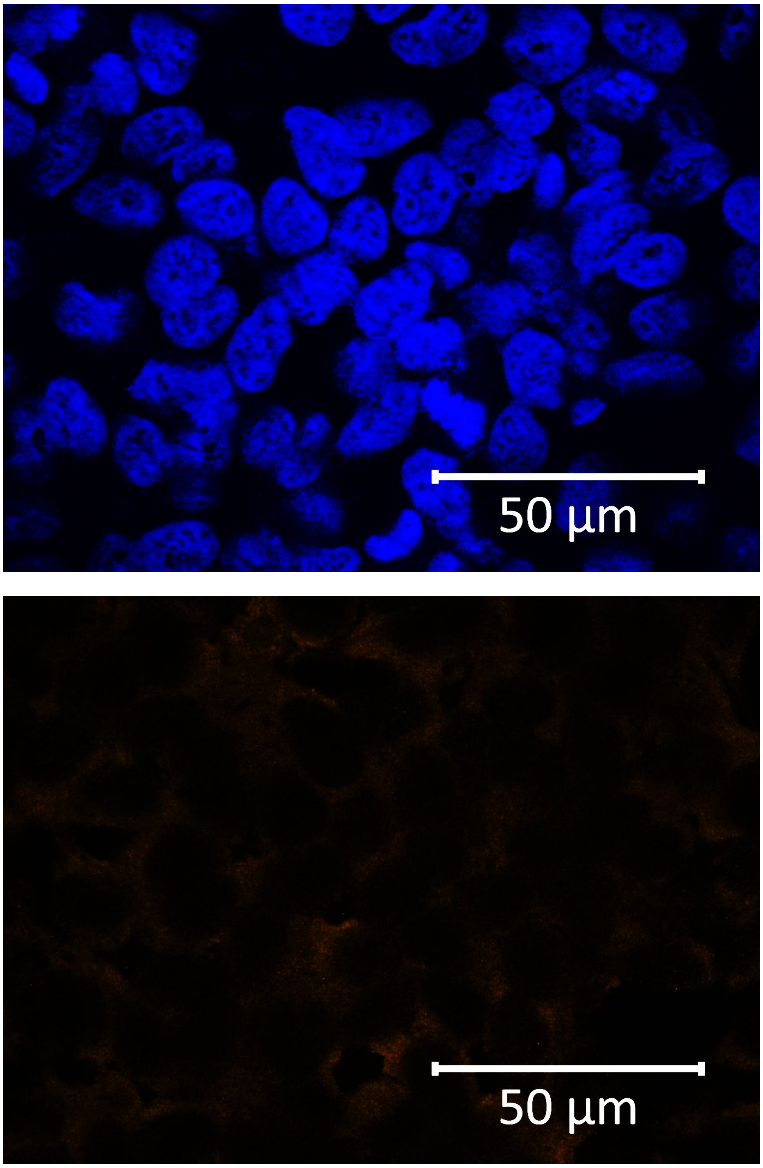

SUPPLEMENTAL FIGURES LEGENDS

Supplemental figure S-1

a: DAPI and NPT2c immunofluorescence in wild-type and mutants NPT2c transfected HEK cells 48 hours after transfection in HEK cells (0.5 µg of DNA and 1 µl of lipofectamine 2000 per well). Florescence localizes mainly at the plasmic membrane in Wild-type NPT2c and phosphate transporting NPT2c mutants. In non-transporting NPT2c mutants, florescence localizes either at the plasmic membrane or not.
b: DAPI and Na,K-ATPase alpha 1 subunit immunofluorescence in wild-type and mutants NPT2c transfected HEK cells 48 hours after transfection in HEK cells (0.5 µg of DNA and 1 µl of lipofectamine 2000 per well). Florescence localizes mainly at the plasmic membrane in Wild-type NPT2c and both phosphate transporting and non-transporting NPT2c mutants.
c: DAPI and NPT2c immunofluorescence in wild-type non transfected HEK cells. Fluorescence signal is weak and does not localize at the plasmic membrane

SUPPLEMENTAL TABLE

Supplementary table 1: ACMG classification criteria used for each variation

Gene	Protein	Variant : nucleotide	Variant : amino acid	Types of mutations	Exon Intron	Protein Domain	ACMG class	ACMG criteria (before)	Minor allele frequency (%)
Variants explored by sodium-dependent phosphate uptake									
SLC34A3	NPT2c	c.241G>A	p.Gly81Ser	missense	4	TM1	2	PM2, BS3, BP4	0.0014
SLC34A3	NPT2c	c.496G>A	p.Gly166Ser	missense	6	IC	4	PS4, PM1, PM2, PP3	0.0039
SLC34A3	NPT2c	c.575C>T	p.Ser192Leu	missense	7	TM3	4	PS3, PM1, PM2, PP5	0.046
SLC34A3	NPT2c	c.781A>G	p.Ser261Gly	missense	8	EC	2	PM2, BS2, BP4	0.027
SLC34A3	NPT2c	c.947C>T	p.Thr316Met	missense	10	EC	3	PM2, BP4	0.019
SLC34A3	NPT2c	c.1046_1047delTG	pVal349Ala fs*243	frameshift	10	IC	5	PVS1, PM2, PP5	0.0012
SLC34A3	NPT2c	c.1208T>G	p.Met403Arg	missense	11	EC	3	PM2, BP4	0.0062
SLC34A3	NPT2c	c.1242C>G	p.Tyr414*	nonsense	12	EC	5	PVS1, PM1, PM2	0
SLC34A3	NPT2c	c.1361A>G	p.Asn454Ser	missense	13	TM6	4	PS4, PM1, PM2, PP3	0
SLC34A3	NPT2c	c.1453C>T	p.Arg485Cys	missense	13	IC	4	PM1, PM2, PM4, PP3	0.054
SLC34A3	NPT2c	c.1454G>A	p.Arg485His	missense	13	IC	4	PM1, PM2, PP3, PP5, BS2, BP6	0.277
SLC34A3	NPT2c	c.1496T>C	p.Leu499Pro	missense	13	TM7	3	PM1, PM2, PP3	0.00082
SLC34A3	NPT2c	c.1585A>T	p.Ile529Phe	missense	13	TM8	2	PM2, PP5, BS2, BP4, BP6	0.274

Abbreviations: PVS1, pathogenic very strong; PS1–4, pathogenic strong; PM1–6, pathogenic moderate; PP1–5, pathogenic supporting; BA1, benign stand-alone; BS1-4, benign strong; BP1-6, benign supporting. To classify variants, we used the rules for combining criteria proposed by the ACMG (Table 5 in: Richards, S. *et al.* Standards and guidelines for the interpretation of sequence variants: a joint consensus recommendation of the American College of Medical Genetics and Genomics and the Association for Molecular Pathology. *Genetics in medicine : official journal of the American College of Medical Genetics* **17**, 405–24, 2015)

Supplementary table 1: continued.

Gene	Protein	Variant : nucleotide	Variant : amino acid	Types of mutations	Exon Intron	Protein Domain	ACMG class	ACMG criteria (before)	Minor allele frequency (%)
Variants not explored by sodium-dependent phosphate uptake									
SLC34A3	NPT2c	c.846G>A	p.Pro282Pro	splice	8	EC	4	PM2,PM3, PP3 (splice), PP5	0.0025
SLC34A3	NPT2c	c.1093+41_1094-15del	p.?		10	intron	4	PM2, PM3, PP3, PP5	0
SLC34A3	NPT2c	c.560+27_561-39del	p.?		6	intron	5	PS4, PM2, PM3, PP3, PP5	0
SLC34A3	NPT2c	c.1717_1732del	p.Asn573Argfs*63	frameshift	13	IC	5	PVS1, PM1, PM2	0
SLC34A3	NPT2c	c.925+20_926-48del	p.?		9	intron	5	PS4, PM2, PM3, PM4, PP3, PP5	0
SLC34A3	NPT2c	c.1571_*80del	p.Leu524_Leu599delins13	in frame	13	IC	5	PS4, PM1, PM2, PM4, PP5	0
SLC34A1	NPT2a	c.1416+3G>A	p.?		12	intron	3	PM2, BP4	1.690
SLC34A1	NPT2a	c.644+5G>A	p.?		6	intron	3	PM2, PP3	0.036
SLC34A1	NPT2a	c.272_292del	p.Val91_Ala97del	in frame	4	IC	3	PS3, BS1	0.00073
SLC9A3R1	NHERF1	c.328C>G	p.Leu110Val	missense	1	EC, subdomain of PDZ1	4	PS3, PS4, PP5	1.498

Abbreviations: PVS1, pathogenic very strong; PS1–4, pathogenic strong; PM1–6, pathogenic moderate; PP1–5, pathogenic supporting; BA1, benign stand-alone; BS1-4, benign strong; BP1-6, benign supporting. To classify variants, we used the rules for combining criteria proposed by the ACMG (Table 5 in: Richards, S. *et al.* Standards and guidelines for the interpretation of sequence variants: a joint consensus recommendation of the American College of Medical Genetics and Genomics and the Association for Molecular Pathology. *Genetics in medicine : official journal of the American College of Medical Genetics* **17**, 405–24, 2015)
